# Commentary: Copper and cuproptosis-related genes in hepatocellular carcinoma: therapeutic biomarkers targeting tumor immune microenvironment and immune checkpoints

**DOI:** 10.3389/fimmu.2023.1265565

**Published:** 2023-08-25

**Authors:** Fangshi Xu, Danrui Cai, Zheng Yang, Jian Yin, Yi Sun

**Affiliations:** ^1^ Department of Urology, Shaanxi Provincial People’s Hospital, Xi’an, Shaanxi, China; ^2^ Department of Ophthalmology, Second Affiliated Hospital of Xi’an Jiaotong University, Xi’an, Shaanxi, China

**Keywords:** cuproptosis, cancer, pathogenesis, bioinformatics, signature

## Introduction

**Figure 1:**
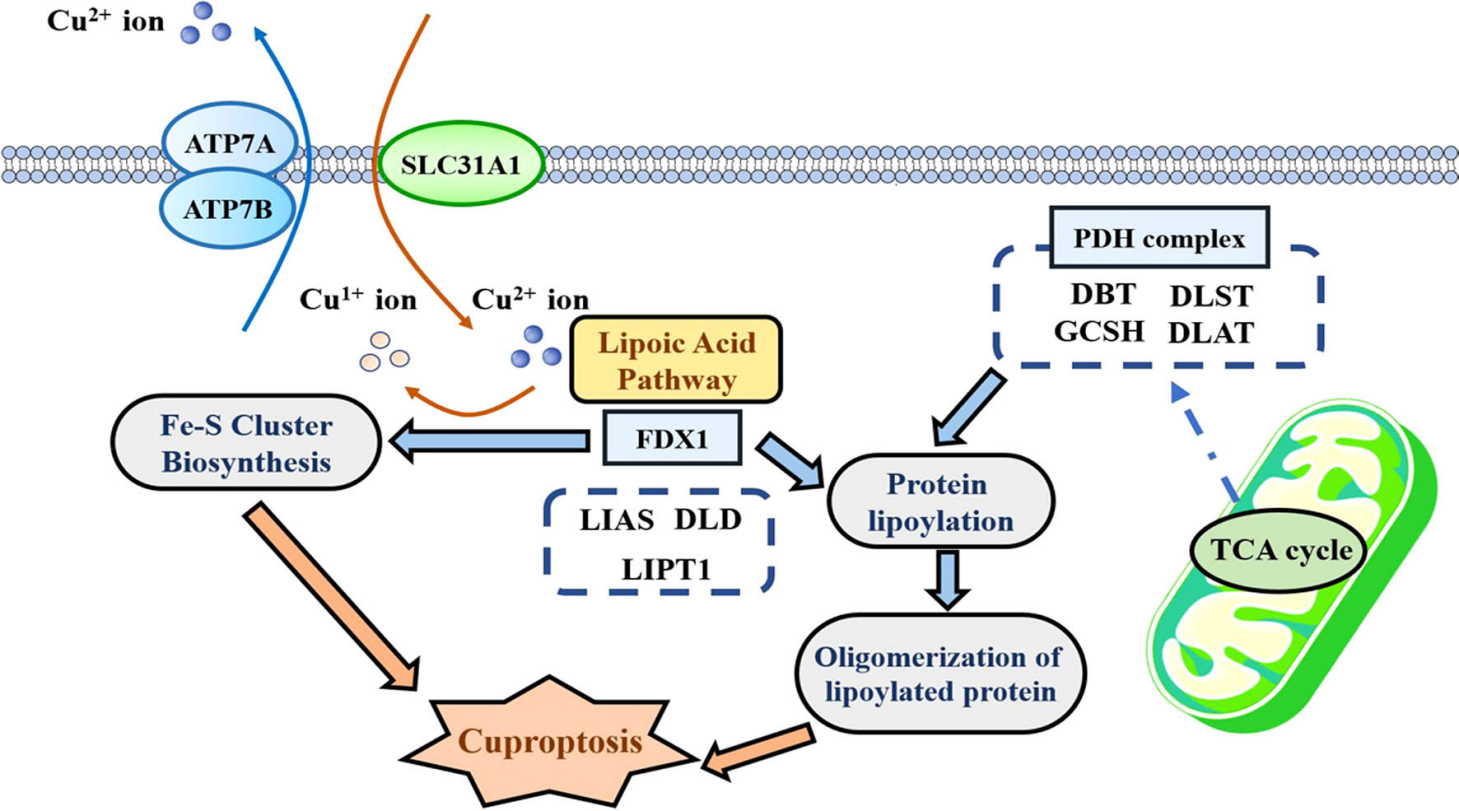
Mechanism diagram of cuproptosis.

Since P Tsvetkov et al. have first coined “cuproptosis” in 2022, this novel pattern of programmed cell death (PCD) greatly expands our horizons of human diseases (1). Mechanistically, FDX1-mediated protein lipoylation and copper-mediated toxic gain drive the onset of cuproptosis (1). As shown in **Figure 1**, FDX1, as a metal reductase, is responsible for reducing Cu^2+^ to its more toxic form Cu^1+^. Next, protein lipoylation is triggered with the aid of FDX1 and six regulators in the lipoic acid pathway. Due to the fact that protein lipoylation is only observed in four enzymes (DBT, DLST, GCSH, and DLAT), all of which participate in the tricarboxylic acid cycle (TCA), the cuproptosis process is subjected to mitochondrial respiration. In the effector phase, copper directly binds to lipoylated protein to increase its cytotoxicity through promoting its aberrant oligomerization. Moreover, copper is able to destabilize Fe–S cluster proteins, thereby enhancing proteotoxic stress. The above-mentioned two processes of copper-induced toxic gain eventually lead to cuproptosis.

Considering the critical roles of other patterns of cell death in cancers, such as apoptosis and ferroptosis, an increasing number of scholars move their attention on the associations of cuproptosis with human cancers. Recently, we focused on the research by X Wang et al. entitled “Copper and cuproptosis-related genes in hepatocellular carcinoma: Therapeutic biomarkers targeting tumor immune microenvironment and immune checkpoints”, which was published in *Frontiers in Immunology* (2). In this study, the authors constructed a cuproptosis-related (CR) signature using Lasso regression analysis to evaluate tumor immune microenvironments (TIMs) and predict the prognosis of patients in hepatocellular carcinoma (HCC). Moreover, they found that some critical CR genes such as PRNP and COX17 were closely related with the expressions of immune checkpoints (ICs), which showed the potentials of the application of a CR model to predict the efficacy of immune checkpoint inhibitors (ICIs).

Despite the great inspiration of their findings to HCC clinical assessment, there is still a long way ahead the clinical application of cuproptosis in HCC. The first issue that needs to be urgently addressed is whether cuproptosis is the dominant pattern of cell death in a specific cancer compared to other PCD. If cuproptosis rarely occurred or is hardly induced in a specific cancer, the corresponding CR signature may be tedious. Moreover, how to detect the intensity of cuproptosis and how to assess the effects of genes on cuproptosis are the other critical issues needed to be addressed. Regretfully, several recent studies in *Frontiers in Immunology* have failed to eliminate the above-mentioned concerns well (3–5). Therefore, we performed the following discussion which aims to provide some insights into further CR research.

## Roles of cuproptosis in cancer pathogenesis: leader or retinue?

As a novel type of PCD, the precise mechanisms of cuproptosis in the onset and progression of human cancers need to be further identified. A bibliometric research revealed that the majority of existing cuproptosis studies only exhibited the bioinformatic functional predictions or associations of cuproptosis in cancer (6). However, how big the roles of cuproptosis in cancer pathogenesis remain elusive. Compared with other PCD, cuproptosis is not characterized by the obvious alteration of cell microstructure—for instance, the abnormal changes in structure of the mitochondria observed through transmission electron microscopy can be the direct evidence supporting ferroptosis, but not for cuproptosis (7). Herein we provided some ideas for the above-mentioned issue.

It is now confirmed that cuproptosis is subjected to mitochondrial respiration due to the fact that inhibitors of the electron transport chain (ETC) as well as inhibitors of mitochondrial pyruvate uptake both hinder the copper ionophore-induced cell death (1). Not only that, the core link of cuproptosis, protein lipoylation, only occurs in four enzymes of the TCA cycle (8). Thus, it is not difficult to surmise that cuproptosis occurrence was tightly related to the activity of the TCA cycle. In the above-mentioned context, we speculate that the activity of the TCA cycle or mitochondrial respiration could act as the indicator to assess the cuproptosis level. At present, there have been reasonable and reliable approaches to detect the alterations of mitochondrial respiration, including the extracellular acidification rate (ECAR), the oxygen consumption rate (OCR), and the detection on the activity of key enzymes or products in the TCA cycle—for instance, X Pei et al. have applied ECAR and OCR assays to confirm the influence of MDH2 on mitochondrial respiration (9). FL Basei et al. have evaluated the changes in the protein expressions of respiratory complexes, such as NDUFB8, SDHB, MTCOI, and SDHB, thereby revealing the regulatory function of Nek4 in mitochondrial respiration (10).

Therefore, it is more reasonable to clarify the cuproptosis level in a certain tumor before constructing a CR signature for cancer clinical assessments. If there are no differences in the activity of mitochondrial respiration, especially the TCA cycle between normal and tumor cells or samples, the constructed CR signature is more like a purely mathematical model rather than an excellent assessment tool related to cuproptosis.

## Paying more attention on the functions of research genes in cuproptosis

To date, a considerable proportion of cuproptosis bioinformatic studies have only investigated the oncogenic or inhibitory functions of CR genes in cancers from the biological perspective, without determining the impact of these genes on cuproptosis—for example, another cuproptosis research published on *Frontiers in Immunology* has established a CR model for predicting a metastatic event in melanoma, and the authors analyzed the effects of FDX1, the core gene in this model, on the proliferation and migration of melanoma cells (11). Similar research strategy is also observed in other studies (12). Nevertheless, the effects of CR genes on the cuproptosis process remain unanswered among these research, which inevitably raises a question on whether these genes actually regulate cancer development through cuproptosis. To resolve this issue, we suggest that researchers could determine the sensitivity of a tumor cell to cuproptosis agonists, such as elesclcomol under the deficiency or overexpression of the target gene. Alternatively, they could ascertain whether cuproptosis agonists are able to reverse the effects of genes on the malignant behaviors of tumor cells.

## Conclusion

The discovery of cuproptosis extremely expands our understanding of cancer pathogenesis and inspires the work enthusiasm of researchers. Of note, conducting scientific functional analysis related to cuproptosis prior to initiating CR research, especially for CR bioinformatic research, is great of significance.

## Author contributions

FX: Writing – original draft, Writing – review & editing. DC: Writing – original draft. ZY: Writing – original draft. JY: Writing – original draft. YS: Conceptualization, Project administration, Writing – review & editing.
